# Experimental pancreatic cancer develops in soft pancreas: novel leads for an individualized diagnosis by ultrafast elasticity imaging

**DOI:** 10.7150/thno.34066

**Published:** 2019-08-14

**Authors:** Nicole Therville, Silvia Arcucci, Aurélie Vertut, Fernanda Ramos-Delgado, Dina Ferreira Da Mota, Marlène Dufresne, Céline Basset, Julie Guillermet-Guibert

**Affiliations:** 1 INSERM U1037, CRCT, Université Paul Sabatier, Toulouse; 2 Laboratoire d'Excellence TouCAN; 3 Laboratoire d'Histologie et d'Embryologie, Faculté de médecine de Rangueil, Toulouse; 4 Service d'Anatomo-Pathologie, IUCT-O, Toulouse

**Keywords:** pancreatic cancer, tumor rigidity, quantitative live imaging, tumor heterogeneity, personalized medecine

## Abstract

Rapid, easy and early pancreatic cancer diagnosis and therapeutic follow up continue to necessitate an increasing attention towards the development of effective treatment strategies for this lethal disease. The non invasive quantitative assessment of pancreatic heterogeneity is limited. Here, we report the development of a preclinical imaging protocol using ultrasonography and shear wave technology in an experimental in situ pancreatic cancer model to measure the evolution of pancreatic rigidity.

**Methods**: Intrapancreatic tumors were genetically induced by mutated Kras and p53 in KPC mice. We evaluated the feasiblity of a live imaging protocol by assessing pancreas evolution with Aixplorer technology accross 36 weeks. Lethality induced by *in situ* pancreatic cancer was heterogeneous in time.

**Results**: The developed method successfully detected tumor mass from 26 weeks onwards at minimal 0.029 cm^3^ size. Elastography measurements using shear wave methodology had a wide detection range from 4.7kPa to 166.1kPa. Protumorigenic mutations induced a significant decrease of the rigidity of pancreatic tissue before tumors developed in correlation with the detection of senescent marker p16-positive cells. An intratumoral increased rigidity was quantified and found surprisingly heterogeneous. Tumors also presented a huge inter-individual heterogeneity in their rigidity parameters; tumors with low and high rigidity at detection evolve very heterogeneously in their rigidity parameters, as well as in their volume. Increase in rigidity in tumors detected by ultrafast elasticity imaging coincided with detection of tumors by echography and with the detection of the inflammatory protumoral systemic condition by non invasive follow-up and of collagen fibers by post-processing tumoral IHC analysis.

**Conclusion**: Our promising results indicate the potential of the shear wave elastography to support individualization of diagnosis in this most aggressive disease.

## Introduction

Pancreatic ductal adenocarcinoma (PDAC) is a dismal disease without effective therapeutic option except surgery. This disease is the most lethal of the common cancers, and is projected by 2030 to be the second highest cause of death due to cancer. The European Union has the highest incidence of pancreatic cancer in the world; incidence is increasing in France, and throughout European latin countries 1, 2. One of the major challenges facing research scientist and the clinical community is the fact that pancreatic cancer is a very heterogeneous disease. In particular, development and evolution of tumors present a great inter-individual variation, as well as significant heterogeneity within the tumor of each individual as assessed by their molecular genetic and genomic characterization 3. Understanding this heterogeneity in a multi-scale integrated manner is vital to enable the newly developed targeted therapies to be efficient 4**.** Heterogeneity is not simply a function of the cancer cells themselves, but encompasses also the manner in which cancer cells interact with other cells of the tumor microenvironment, also called stroma**.** This, in turn, impacts the cancer response to treatment therapies, which is different in each individual. As a consequence, treatment choice and its efficiency evaluation should be individualized and tested as such in preclinical settings, to help clinicians predict the best therapeutic option in the shorter timeframe possible, and then to assess the variablity of response of the newly developed therapeutic strategies 3. However, easy and predictive methods to assess tumor tissular composition, therapeutic options and early treatment efficiency are lacking both in clinical and preclinical settings.

Ultrasound echography (US) is an inexpensive, non-invasive method of diagnostic or treatment evaluation that can be performed easily and repeatedly. However, US images do not quantitatively measure the changes of the physical characteristics of the tumors, possibly indicative of the changes of tumor physiopathology, neither measure the physical changes occuring in pancreatic parenchyma before the detection of the tumors, which could help to diagnose earlier 5.

Two dimentional shear wave elastography (2D SWE) as opposed to point shear wave elastography (point SWE) is a recent live ultrafast imaging method which allows a longitudinal follow-up of tissue rigidity in time and space 6-8. The propagation of shear waves in the tissue correlates to tissue elasticity and the wave velocity is proportional to tissue elasticity 6. The propagation velocity of transverse shear waves in human liver fibrotic tissue is higher than in healthy liver parenchyma 7, 9. Interestingly, this value is measured without applying a constraint on the tissue when imaging and without dissection as described in other studies using xenograft tumors 10, 11, including from pancreatic cancer 12, 13 .

Tissue stiffness affects tumor growth, invasion, metastasis and treatment. Currently, several elastography procedures exist and measure tissue stiffness 7, 14, 15-17. While used in preclinical models such as subcutaneously injected tumors 18, 19, the assessment of experimental *in situ* intra-abdominal tumors, and in particular of pancreatic tumors is emerging (20, 21, with ultra fast 2D-SWE technology 20, 22. Others recently performed such measurements *ex vivo* after organ dissection in a genetically engineered mouse (GEM) model of colon cancer 23*.* As opposed to point measurements by other technologies, the real‐time imaging by 2D-SWE allows in theory the assessement of the stability of the measurement and quantification of an average value of rigidity in a large region of interest for higher reliability.

We aimed to further investigate the role of ultra fast shear wave elastography as a complement to B mode echography using a spontaneous complex mouse model of PDAC, so as to set a standard of this physiologically relevant quantitative imaging of pancreatic cancer development. Here, we report on multimodal echographic and elastographic non-invasive imaging in GEM with endogenous aggressive PDAC to demonstrate that its intratumoral and interindividual heterogeneity in rigidity is associated with different tumoral evolution.

## Results

### Besides mimicking human pathology, KPC mice model the heterogeneous development of pancreatic tumors

**Figure 1:**
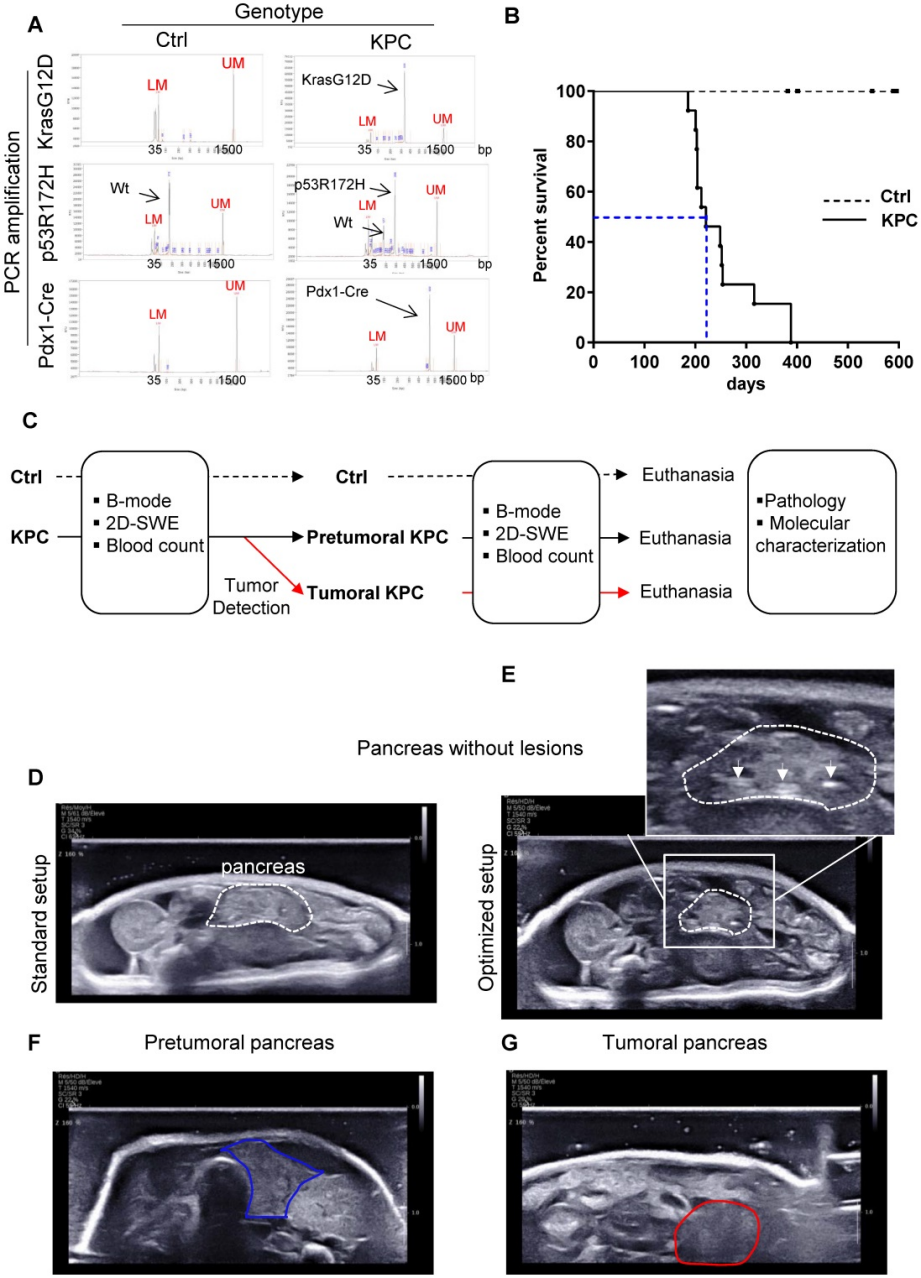
** Besides mimicking human pathology, KPC mice model the heterogeneous development of pancreatic tumors. A:** Result of genotyping of triple transgenic mice using capillary electrophoresis analysis. Internals markers show a peak at 35 bp (Lower marker LM) and 1500 bp (Upper Marker UM) in all samples. **B:** Day survival curves of control (Ctrl: dashed line) and *LSL-Kras^G12D/+^*;*LSL- Trp53^R172H/+^*;*Pdx-1-Cre* (KPC: continuous line) mice. Kaplan-Meier curves reveal a median survival in triple mutant mice of approximately 6 months (blue dashed line). (n=13) **C:** Experimental setting to study the evolution of pancreas rigidity in Ctrl and KPC mice. Imaging is coupled to clinical and biological (blood parameters) assessment. Macroscopic and microscopic analysis of pancreas and known sites of metastasis (peritoneal cavity, liver, lung, spleen) is performed at end point (pathology). KPC mice with tumors are euthanized according to ethical requirements. Both Ctrl and KPC mice were euthanized at maximum 53 weeks, even if no tumor was detected in KPC genetic background (n=3). **D-G:** B-mode ultrasound representative images obtained on pancreas without lesions (pancreas being the region of interest circled in white dashed lines) and pancreatic tumor (circled in red) with standard setup (D) or optimized setup (E, F and G). White arrows show hyperechogenic striated areas in normal pancreas. In E, these features disappear (circled in blue), but no tumor is detected.

Triple transgenic mice with intrapancreatic mutation of KrasG12D and p53R172H (Pdx1-Cre^+^/- and LSL-Kras^G12D/+^ and LSL-Trp53^R172H/+^ mice, called hereafter KPC) mice were used as a model of locally advanced PDAC that exhibits a typical human-like morphology with abundant desmoplasia, moderate to poor epithelial differentiation and a highly aggressive clinical course (Figure 1 A, B; for weight curve Supplementary Figure 1) 24, and was followed by the newly developed Aixplorer (7-22 MHz probe) (Figure 1C). This model also progressively develops the preneoplastic lesions prior tumor development albeit at different kinetics in each individual. As previously shown in this model, lethality is gradual, with a medium survival of 221 days, associated with different kinetics of tumor detection (Figure 1B). An optimized setup allowed the detection of tumors using Aixplorer (Figure 1 D-G, Supplementary Method, Supplementary Figure 2 -3, Supplementary Table 1). Tumor detection range was from 26 to 35 weeks. We find that Aixplorer allowed detection at the smallest volume of 0.029cm^3^. This imaging method did not allow the detection of preneoplastic lesions based on echographic images. We however found that the loss of striated hyperechogenic areas is an early sign of pancreatic parenchyma modification in both settings, possibly due to defects in normal ducts (Figure 1E as compared to Figure 1D). This observation is dependent on the training of the observer and is not observed in each mice; hence, we next aimed to develop a quantitative method to analyse early modifications in pancreatic parenchyma.

### A post-processing quantification is necessary to reproducibly measure elastography in the pancreas

**Figure 2:**
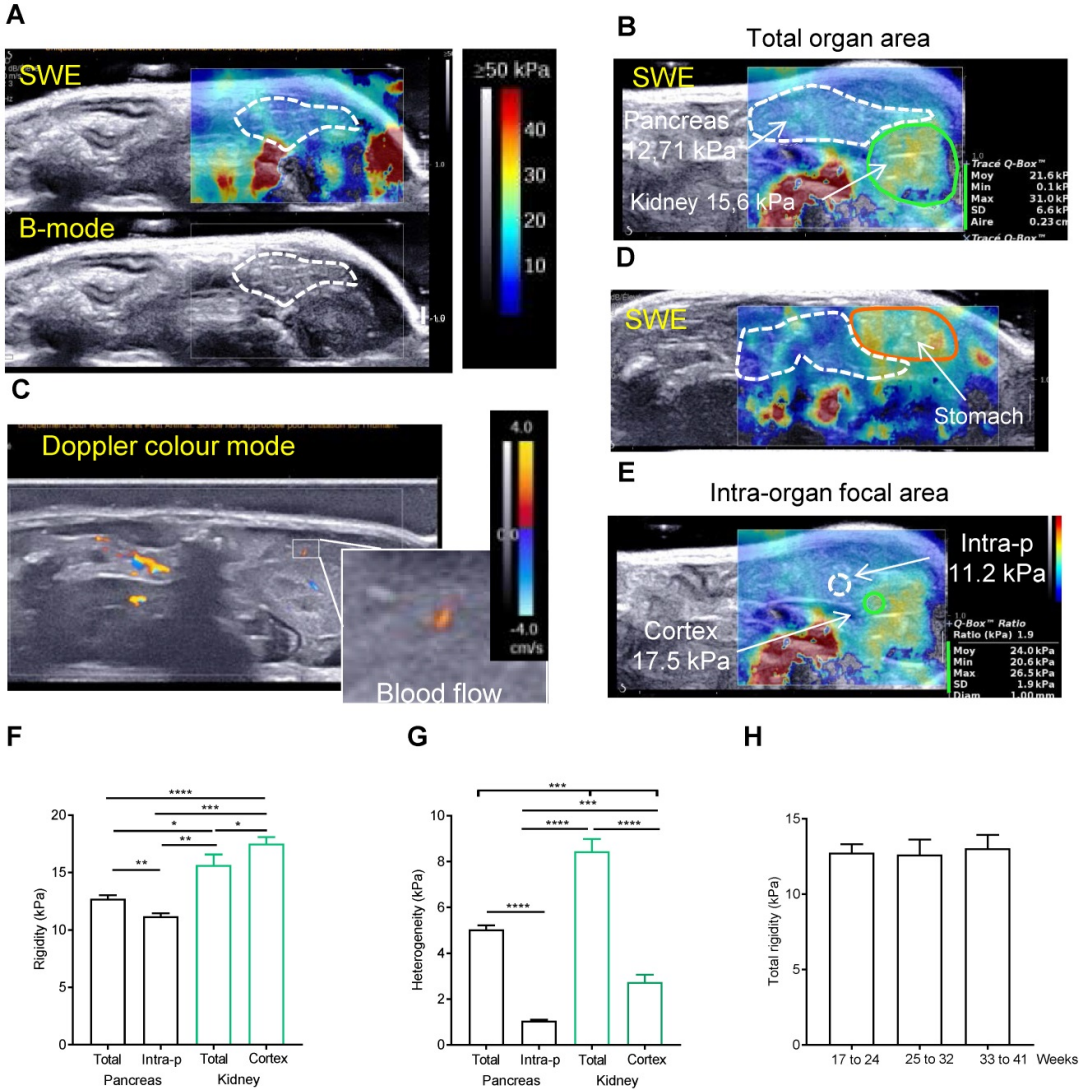
** A post-processing quantification of a representative area is necessary to reproducibly measure elastography in the normal pancreas. A-E:** Side-by-side display of anatomical B-mode US image (grey image, bottom) and an elastography image restricted to the set study box (colored image, top) obtained with echographic B-mode and 2D-SWE mode, coupled to doppler mode. Red color represents stiff tissue and blue color reflects soft tissue (scale ≥50kPa-0kPa as shown in A). A,B show representative image of pancreas without detected tumors in Ctrl mice with two sector sizes. C represents a Doppler image of the image shown in B. Color scale ranges from -4 to 4 cm/s. D shows how the adjacent organs influences the rigidity of the pancreas. E represents an image showing how intrapancreatic and cortex kidney rigidity measurements are set in pancreas without detected tumors. White dashed line: pancreas limit determined with B-mode. Green line: kidney limit. orange line: stomach limit. In B and E, an example of measurements is shown including the mean rigidity (Moy), the minimal (Min) and the maximal (Max), the standard deviation of the values measured in the set zone (SD) and the area or the diameter of the measured zone (Aire or Diam). The focal zone is set at 1 cm ± 0.2 cm. **F, G, H:** Rigidity and its heterogeneity (measured with the SD of the measured area) is plotted as a mean of raw mean area values ± SEM from 8 mice measured from 17 to 41 weeks (F, G) or at week intervals (H) (n=8 mice; see Supplementary Table 2 for all raw data). Paired t-test test: ***:p<0.001, ns: p>0.05.

We then used the elastography module of Aixplorer technology. The elastography measurements were carried out from 17 to 41 weeks old in 8 Ctrl mice. Success rate of imaging both pancreas and kidney in full was not 100%; in most often case, it was due to a swelling of the stomach (Supplementary Figure 2). If not possible, the imaging was repeated the next week. To ensure a repeatability of the measurements, pancreas were imaged at a constant focal zone of -1cm, with left kidney as a reference organ. The elastograms of pancreas measured in Pascals (Pa) are heterogeneous in time and space, with measured point values ranging from 4.7 to 24.6kPa (Figure 2A for normal pancreas). For each setting, see colour chart ranging from 0 (Blue) to ≥ 50 kPa (Red) and the elastogram represented in colour (top) superposed to the echographic B-mode image (bottom). A normal pancreas presents low intensity values (color coding is <30 kPa) with peaks in rigidity (with color coding >30 kPa) (Figure 2B). These measured elastograms were then post-processed to quantify *in vivo* rigidity. When we first performed tracing measurements that encompassed the entire organ (total organ area) (Figure 2B), values were found heterogeneous. Indeed, the presence of red zones in the borderline of the organ are included in the total organ area measurements as shown in Figure 2B (white values) and increases the standard deviation of the measure, SD being representative of the heterogeneity. While this could be an artefact generated by shear wave reflection, we find, on the image shown in Figure 2B, that it was explained by the influence of arteries and veins as shown in Figure 2C in doppler mode. Zones which are in contact with stiffer organs such as the stomach also present higher values (yellow colouring observed in pancreas corresponding to values range of 30-35kPa) (Figure 2D). To confirm our finding, we compared these data to the rigidity in predefined quantitative boxes of 1mm in diameter set at distance from the organ margin and blood flows, measure called intra-pancreatic area. The measure of the reference organ acquired in the same image at the same focal zone was also performed (total kidney area, kidney cortex area)(Figure 2E). The intra-pancreatic measure of the pancreas was taken at distance from other organs and blood vessels on the live image in SWE mode. The average rigidity of the total or intra-pancreatic pancreas and of the total kidney or kidney cortex is 12.71kPa ± 0.33, 11.19 kPa ± 0.26, 15.66 kPa ± 0.91 and 17.54 ± 0.55 kPa, respectively (based on the average of all images acquired in longitudinal) (Figure 2B,E,F). As expected, intrapancreatic measure was statistically less heterogeneous than the total pancreatic area as measured by the mean SD of these values (Figure 2G); the average total rigidity was significantly different from the intra-pancreatic rigidity. Kidney cortex is statistically more rigid than pancreas (total area and intra-pancreatic area), as observed with the elastograms shown in Figure 2 A, B, E (green zone below the pancreas). Of note, the total kidney area had a higher heterogeneity value due to the heterogeneous internal areas (medulla) of this organ; kidney being more internal, the full assessment was not possible in all cases leading to the absence of elastogram in its deeper part (represented on the elastogram as grey zones, Supplementary Figure 2A). We confirm that both total pancreatic area and intra-pancreatic area measurements led to measurements of pancreas in accordance to the representative elastograms (Figure 2A,B,E). The measure of total rigidity is stable in time (Figure 2H).

Total area, intra-pancreatic area of the pancreas, kidney cortex measurements lead to reproducible and consistent quantification of normal *in situ* tissular rigidity.

### Longitudinal measure of rigidity in Kras and p53 mutated pancreas reveals the inter-individual heterogeneity of tumors developed *in situ* at detection and during evolution*.*

**Figure 3:**
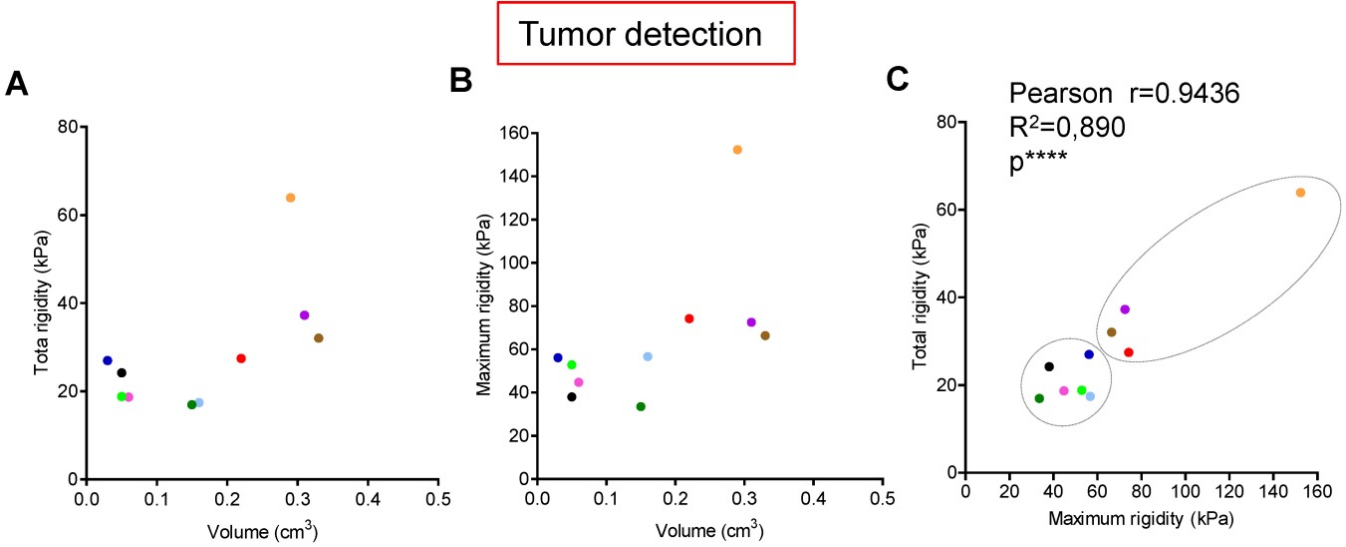
** Longitudinal measure of rigidity in Kras and p53 mutated pancreas reveals the inter-individual heterogeneity of tumors developed *in situ* at detection*.* A-C:** Individual measurements of rigidity (total tumoral, maximal tumoral) and volumes at detection are shown (n=10; individual values, also see Supplementary table 2 for all values). Pearson's correlation test were significative for C (****, Pearsons r=0.9436; R^2^=0.890).

Tumors are known to present an increase of rigidity. We questioned if quantitative measurement of rigidity in pancreas could help to detect *in situ* developed tumors. We performed tumor detection using B-mode imaging in a cohorte of KPC mice, and longitudinally analyzed body weights and elastography in pancreas with SWE mode (Figure 1C). SWE mode did not allow to detect tumors earlier; however, the diagnostic of tumor was confirmed by an increase of rigidity. Total tumoral rigidity and maximal intratumoral rigidity point value were not significantly correlated with tumor volume at detection (Figure 3A,B). Total tumoral rigidity was correlated with maximal rigidity value (Figure 3C). Analysis of total tumor rigidity as a function of maximal rigidity value individualized a group of tumors with lower total and maximal rigidity (Figure 3 C).

We then followed the elastogram of five tumors for which we had values at detection and at sacrifice (corresponding to the ethical limiting point), two in the lower rigidity group (blue, pink), three with high rigidity values (red, orange, purple). Blood perfusion is known to influence the rigidity values 25. We confirmed using the Doppler imaging mode that pancreatic tumors developed *in situ* were not perfused (Supplementary Figure 4); in humans, pancreatic tumors are known to be unperfused. We showed that tumors from KPC mice presented heterogeneous intratumoral elastograms both at detection and sacrifice as shown in Figure 3A,B (point values ranging from 18.5 to 166.1kPa). Interestingly, we also observed an inter-tumor heterogeneity (Supplementary Figure 5). The global rigidity of the tumors increased between the two time points for the 5 KPC mice. However, we also observe an heterogeneity in the evolution of total and maximal rigidity, which with the size of our cohorte was not associated with changes in survival. Further work is needed to define quantitative markers of tumoral heterogeneity as a possible method to discriminate a type of experimental pancreatic tumors.Experimental *in situ* PDAC presents a great inter-individual variation, as well as significant heterogeneity within the tumor of each individual. The rigidity area appears as a possible method to discriminate a subtype of experimental pancreatic tumors.

### Longitudinal measure of rigidity in normal, genetically altered pretumoral pancreas and tumors reveals a decrease of rigidity before tumor detection

**Figure 4:**
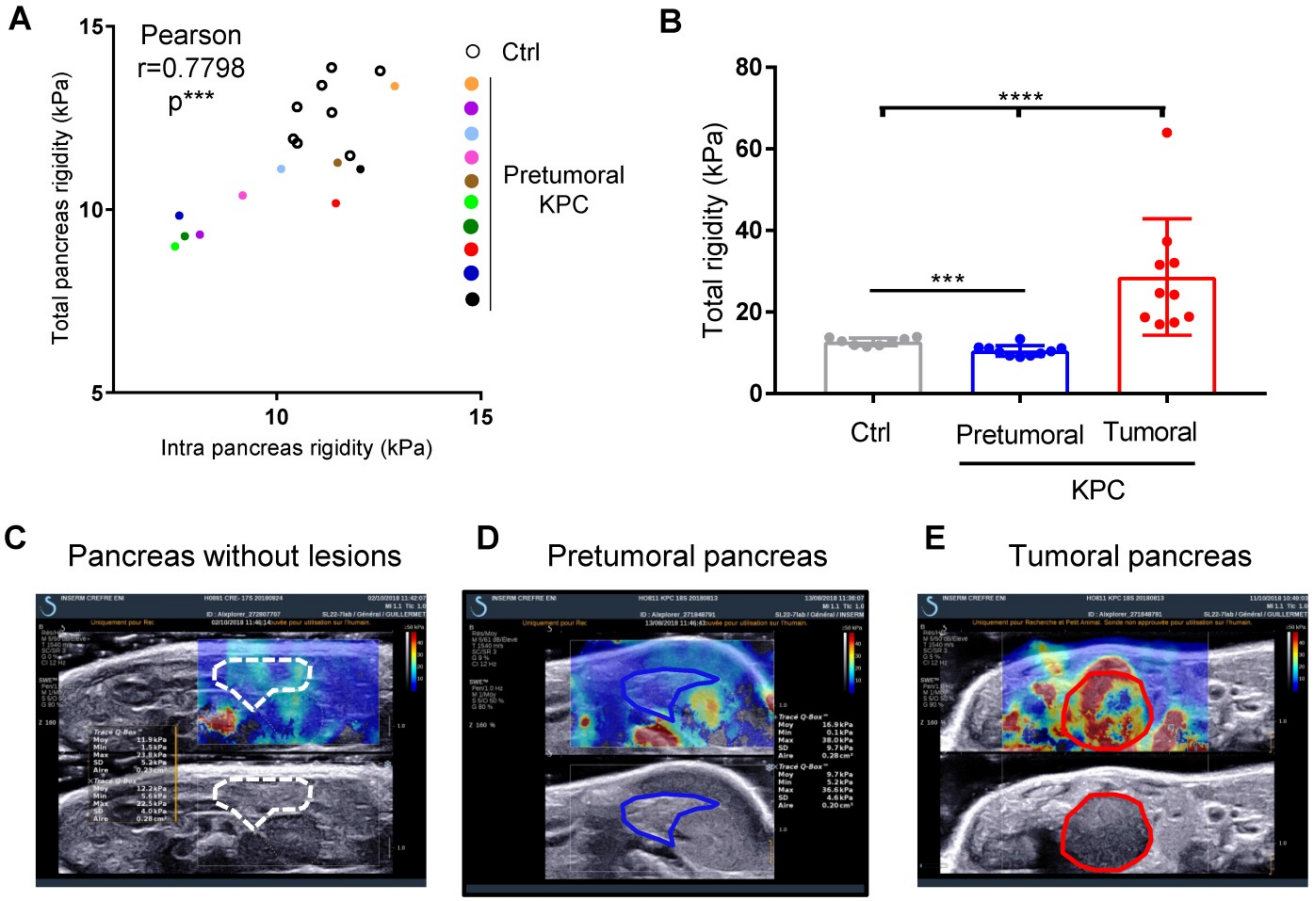
** Longitudinal measure of rigidity in normal, genetically altered pretumoral pancreas and tumors developed *in situ* reveals a decrease of rigidity before tumor development. A:** Elastographic measurements in Control (n=8) or KPC mice (n=10) prior tumor detection (pretumoral KPC). Data represent individual mean values of each mouse. **B:** Mean values of pancreatic rigidity in kPa. Data represent mean ± SEM. Mann Whitney: *: p<0.05; **: p<0.01; ***: p<0.001 Control (n=8) or KPC mice (n=10) **C-E:** representative images of a Ctrl mouse and of the same KPC mouse before and after tumor detection.

We next questioned if the measure of pancreatic tissular rigidity could detect the microscopic changes in the pancreatic parenchyma which occur before tumor development, possibly helping in an earlier detection of tumors. Indeed, pancreatic oncogenesis develops from preneoplastic lesions, encompassing acino-ductal metaplastic lesions (ADM), pancreatic intraepithelial neoplasia (panIN) or cysts (mucinous cystic neoplasm (MCN), intra-ductal papillary mucinous neoplasm (IPMN). Very frequently (> 90% of cases), and at a very early stage (from low grade panIN), the activating mutation of the Kras oncogene is found. These lesions are found in KPC mice. We thus longitudinally analyzed body weights and elastography in pancreas with SWE mode, while tumor detection was performed using B-mode imaging (Figure 1C). We compared pancreatic elasticity after 18 weeks (Figure 4A). Pretumoral KPC aged > 18 weeks presented a baseline rigidity of total and intra-pancreatic parenchyma with decreased values compared to Ctrl mice (Figure 4A, B). In time, KPC pancreas elasticity significantly decreased (Figure 4B). Hence, pro-tumorigenic mutations influence pancreatic elasticity prior to tumor detection. This alteration of pancreatic baseline elasticity preceded the increase in rigidity observed in detected tumors (Figure 4C, D, E for representative images; Supplementary Figure 6 for individual values in time).

Ultra fast shear-wave elastography allows the quantification of a pretumoral stage in a reproductive manner and demonstrates in a non invasive manner the increase in mechanical rigidity in tumors.

### Markers of senescence are present in the pancreatic pretumoral niche, where increased elasticity was measured

**Figure 5:**
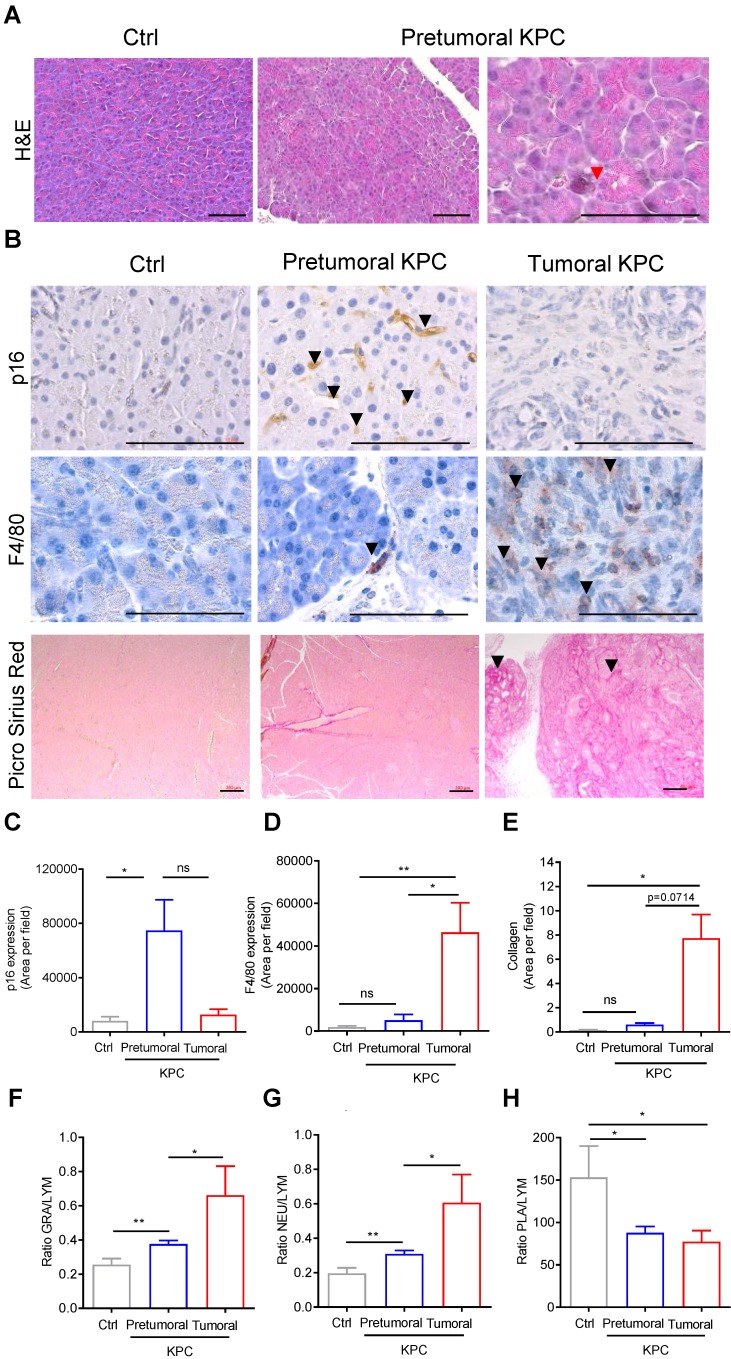
** Markers of senescence are present in the pancreatic pretumoral niche, where increased elasticity was measured. A-B:** H&E, IHC using indicated antibodies & picrosirius red analysis of pancreas (Ctrl, pretumoral or KPC at euthanasia). Scale: 200µm. Red arrows represent senescent cells. Black arrows represent positive DAB (p16) or AEC (F4/80) or picro sirius red stainings. **C-E:** Quantification of stainings in B (mean area per field in ≥ 3 images at 63 x) are presented as mean ± SEM. n=3 in each group. Mann Whitney test: *: p<0.05; **: p<0.01; ***: p<0.001. **F-H:** Circulating granulocyte, neutrophil, platelet values represented as ratios to circulating lymphocytes. Mean± SEM. n≥3 in each group Mann Whitney test: *: p<0.05; **: p<0.01; ***: p<0.001.

We then aimed to discover the origin of the baseline modification of elasticity induced by protumoral mutations in the pancreas prior tumor development (Figure 4), by analyzing the histology of the pancreas (Figure 5A). In the pretumoral KPC, we could not detect the presence of preneoplastic lesions. Because we did not analyzed the whole pancreas, we cannot completely refute their presence. Instead, we detected the presence of acinar cells with collapsed morphology, possibly corresponding to senescent cells (Figure 5A, red arrow). To confirm the presence of senescent cells only in this condition, the representative cytoplasmic anti-p16 senescent marker was found only in pretumoral condition as shown in Figure 5B, black arrows. This staining is reminiscent to the type of staining previously observed only in preneoplastic lesion by others 26. A significant increase of p16 staining area was detected only in pretumoral KPC as compared to Ctrl (Figure 5C).

Finally, we searched whether this modification in rigidity of the tissue could be correlated with changes in stainings of F4/80 macrophages, in picro-sirius red positive collagen fibers, or in circulating white blood cell parameters, inflammation being usually associated with senescence in pretumoral condition. For the latter, we analyzed blood counts in the cohorts of mice defined above in Figure 1C and plotted granulocyte / lymphocyte (GRA/LYM), neutrophil / lymphocyte (NEU/LYM), and platelet / lymphocyte (PLA/LYM) ratios. We could detect a significant difference in these three parameters in the pretumoral condition; F4/80, picro-sirius red positive stainings, GRA/LYM and NEU/LYM ratios further increased in tumors as compared to Ctrl (Figure 5B, D-G).

Microscopic tissular modifications in the pretumoral tissue where the cancer originates, tissue also described as the pretumoral niche, relate to elastographic measurement modifications. Further modifications of rigidity in tumors is associated with changes in the tissular composition (inflammatory infiltration, collagen deposits). Measure of apparent tissular rigidity by SWE appears as an efficient mode to non-invasively qualify and quantify tissular composition parameters in both pretumoral niche and in locally advanced primary tumors of the pancreas (Graphical Abstract).

## Discussion

Advances in therapeutic approaches and molecular subtyping of PDAC 27-31 impose the necessity for individualized early therapeutic diagnostic through an early evaluation of therapy effects in this often rapidly progressing disease. Early therapy-induced effects are probably mirrored in tissue reorganization, changes in number of tumoral and stromal cells (cellularity), cell size or shape, matrix deposition, immune cell recruitment, while measurable changes in tumor volume become apparent at later time points (if any in this pathology). Several novel therapeutic strategies aims at modifying the differentiation of the lesions or the tumoral stroma to modulate their aggressiveness 3. Hence, novel quantitative imaging methodologies based on the physiological changes of the tumors and not based on their volume evolution only will be necessary to assess their early action.

The pancreas is a small organ located deep into the body and is strongly affected by aortic pulsation, which complicates the imaging possibilities 5. Increased rigidity is reproducibly observed in tumors as well as in fibrotic pathologies as measured by others using this method or other technological approaches 12, 13, 18, 19, 21, 32, 33. Our data favorably argue that ultrafast shear wave elastography is an efficient method to characterize the biophysical properties of pancreas prior tumor development as well as pancreatic tumors in correlation with microscopic changes, rendering this technology an attractive, easy and cheap option to detect pretumoral condition, diagnose tumors and follow therapeutic intervention in basic and preclinical settings.

Surprisingly, in our model, pretumoral niche was associated with a significant baseline lower rigidity in the pancreatic parenchyma. Our data so far show that elastography detects earlier signs of tumoral development induced by focal oncogenic mutations. These signs appear to be early pancreatic inflammatory parenchyma (as assessed by increased senescence), translated as a decrease in tissue rigidity. *In vitro* data show that cancer cells present an increased elasticity possibly due to changes in their cytoskeleton properties 34, 35. Because we previously showed that inflammatory conditions in an oncogenic background induces actin cytoskeleton remodelling in exocrine acinar cells and that this intracellular alteration constitutes an early and necessary step in pancreatic lesion formation 36, we speculate that this increase in elasticity found in pancreatic parenchyma is related to a change in actin cytoskeleton polymerisation, indicative of the early stage of cell transformation.

The biomechanical properties of a tissue in terms of elasticity, relatively measured as a rigidity parameter in Pa vary markedly between organs and tissues, and are inherently related to tissue function and content. Increased expression of enzymes modifying extracellular matrix (ECM) by stromal cells results in increased collagen linearization and tissue rigidity in pre-malignant breast tissue 37, rigidity appears to favor tumoral progression 18, 33, while stroma remodeling enzymes such as LOX (Lysyl oxidase) favor metastasis and drug resistance in pancreatic cancer 38. Surgery-induced matrix softening increases the risk of metastasis 39. Further to these findings, our knowledge of the evolution of tumoral mechanical characteristics needs now to be increased to understand the determinants of the impact of this heterogeneous increase in rigidity. Most studies measuring tumoral rigidity describe an increase in these properties which is radially homogeneous within the different layers of the modelled tumors 10; most live mapping of tumor rigidity is made in heterotopic and orthotopic xenografts models 25. These models show that the tissue where the tumoral cells are implanted influences the rigidity measurements 25, further stressing the importance of studying tumor mechanical properties in tumors developed *in situ* 11. The intrinsic co-development of tumors and of the target organ tumoral niche is likely to influence tumor mechanical properties.

Finally, survival of patients with PDAC does not correlate with total fibrillar collagen content within the tumor, but is significantly linked to the localized increased thickness of collagen fibers directly adjacent to PDAC epithelium 32, leading to a local increased rigidity. Hence, a refined spatial mapping of tumoral and tissular mechanical characteristics could be of importance to determine their clinical relevance.

In conclusion, early signs of tumor development include changes in the mechanical properties of tissues. As tumors metastasize, some cancer cells within stiffen their microenvironment by increasing the production and cross-linking of collagen. This local rise in rigidity helps the metastatic cells migrate and invade other regions of the body, this occur in coordination with the recruitment of protumoral immune cells. Our data pave the road for the development of novel preclinically and clinically relevant methodologies to assess tumor initiation and tumor - environment heterotypic dialog in pancreatic cancer.

## Material & Methods

### Animals and Ethical requirements

The LSL-Kras^G12D^ and LSL-p53^R172H^ knockin (from D Tuveson, Mouse Models of Human Cancers Consortium repository (NCI-Frederick, USA), Pdx1-cre (from DA Melton, Harvard University, Cambridge, MA, USA) strains were interbred on mixed background (CD1/SV129/C57Bl6) to obtain compound mutant LSL-Kras^G12D^;LSL-p53^R172H^;Pdx1-Cre (named KPC). Littermates not expressing Cre as well as Pdx1-Cre of the same age were used as control. All procedures and animal housing are conformed to the regulatory standards and were approved by the Ethical committee according to European legislation translated to French Law as Décret 2013-118 1st of February 2013 (APAFIS 3601-2015121622062840). Genotyping was performed using primers as described in 36 and analyzed with Fragment analyzer instrument (AATI) with dsDNA 910 Reagent kit, 35-1500bp (AATI). Blood counts (from EDTA tubes Microvette #16.444, SARSTEDT AG & Co) were performed using Yumizen H500 hematology analyzer (HORIBA), calibrated with murine blood.

### Imaging procedure

KPC and control mice (Ctrl) are imaged once a week. The probe SuperLinear**™ SL22-7lab** (Supersonic Imagine)with preset called "standard" or "optimized" (Supplementary Table 2) was used. After echographic detection of the pancreas, a study box is placed and a US wave is applied focused at different depth, compressing the tissue. The compression of the tissue generate shear-wave perpendicular to US axis, which are measured in live with SuperSonic Aixplorer (France). Colourimetic maps corresponding to the measurement of these waves are immediatly available. Elasticity (E) is measured in kiloPascal (kPa), deducted from the velocity of shear waves (E=3ρV_c_^2^) in the studied region of interest. Measurement of the shear wave speed results in qualitative and quantitative estimates of apparent tissue elasticity. Tumor volume is calculated as follows: Width^2^ x Length x 0.5. Mean detection time of tumor is of 29 weeks; hence this time point was considered as T0 for the Ctrl cohorte. Rigidity values corresponds to the mean of E measured one to six times at each time point. During post-processing analysis, images in the pretumoral of Ctrl pancreas with grey zones were excluded, because values are repeated in time. Some tumors presented limited areas (<5-10%) with grey zones, images which could not be improved by changing the angle of imaging; they were not excluded.

### Histology and Immunostaining

Hematoxylin eosin stainings were conducted using standard methods on formalin-fixed, paraffin-embedded tissues. All pancreata were analyzed in blinded fashion. Pancreas were fixed in 10% neutral buffered formalin and embedded in paraffin. For histopathological analysis, pancreata were serially sectioned (4 µm) and every 10 sections stained with hematoxylin and eosin. Histopathological scoring of pancreatic lesions was performed using serial H&E-stained sections (100 µm apart, 2 sections per pancreas). Picro sirius red staining was performed using the manufacturer condition (AbCAM#150681), with minor modifications corresponding to a 30 minute only incubation with the staining reagent.

Immunostainings were conducted using standard methods on formalin-fixed, paraffin-embedded tissues. Antigen retrieval and antibody dilution (p16, F12 clone, Sigma #sc-1661, 1/100, citrate antigen retrieval; F4/80, Cl A3-1 clone, Pierce #MA1-91124, 1/100, Proteinase K antigen retrieval) was carried out as described in table below followed by AEC or DAB incubation prior secondary antibody (ImmPress (MP-7402, Vector) or BA-4001 (1/50, Vector)).

### Statistics

Experimental data provided at least 3 experimental replicates. Statistical analyses were performed with GraphPad Prism using t-test, or parametric Mann-Whitney tests: * p < 0.05, ** p < 0.01, *** p < 0.001. Non-significant (ns) if p ≥ 0.05. Correlation analysis was performed using Pearson r test. Statistical relevance of the cohort size was determined using a Power Calculation test (www.lasec.cuhk.edu.hk/.../power_calculator_14_may_2014.xls).

## Supplementary Material

Supplementary materials, procedure, figures and supplementary table 1.
