# Supplementary material for: Experimental pancreatic cancer develops in soft pancreas: novel leads for an individualized diagnosis by ultrafast elasticity imaging
Source: Theranostics. 2019 Aug 14;9(22):6369–79. doi: 10.7150/thno.34066 (PMC6771236; doi:10.7150/thno.34066)
Supplement: Supplementary file 1 — Supplementary materials, procedure, figures and supplementary table 1. [file thnov09p6369s1.pdf]

Experimental pancreatic cancer develops in soft pancreas: novel leads for an individualized diagnosis by ultrafast elasticity imaging.

Therville N*[1,2], Arcucci S[1,2], Vertut A[1,2], Ramos-Delgado F[1,2], Dina Ferreira Da Mota[3], Dufresne M[1], Basset C[1,2,3,4], Guillermet-Guibert J[1,2].

[1]INSERM U1037, CRCT, Université Paul Sabatier, Toulouse;
[2]Laboratoire d'Excellence TouCAN;
[3] Laboratoire d'Histologie et d'Embryologie, Faculté de médecine de Rangueil, Toulouse;
[4] Service d'Anatomo-Pathologie, IUCT-O, Toulouse.

Supplementary Legends

Supplemental Figure 1: **Weight curves of Ctrl and KPC cohorts.** Data comparing the mouse weights of KPC and Ctrl mice across time. n>8 in each group.

Supplemental Figure 2: **Troubleshooting for pancreatic 2D SWE quantification and normalisation. A:** SWE mode representative image of an absence of measure in the reference organ, kidney. **B:** SWE mode representative image of difficulty to image the tumors, which changes the anatomy of the abdomen. The reference kidney at the latest time points is difficult to image on the same frame than the tumor, preventing the normalization. **C:** SWE image with artefacts (color gradient outside the mouse). **D:** B-mode image of the stomach causing a shadow on the pancreas and left kidney.

Supplemental Figure 3: **Specific details linked to Aixplorer use in small animals. A-C:** Equipment required for the experimental setup. **D-E:** Positioning of the mouse. **F:** Aixplorer control panel. Also see supplemental methods.

Supplemental Figure 4: **Representative Doppler image of KPC tumors.**

Suppl Figure 5: **Intra-tumoral rigidity is heterogeneous and leads to different tumor evolution patterns.** Representative images of KPC tumors at detection and at euthanasia using maximal visualisation size.

Suppl Figure 6: **Evolution in time of individual rigidity values.** Control (n=8); KPC mice (n=10).

Supplemental Table 1: **Parameters of optimized settings adapted to internal abdominal organs.**

Supplemental Table 2: **Raw data and analysis of Ctrl and KPC mice in time.**

Supplementary methods for the use of Aixplorer apparatus adapted to small animals

Materials

Specific Reagents :

- Experimental murine models of PDAC. The use of animals for these experiments requires the approval of Ethics Committee or an equivalent regulatory organization.

- Isofluorane (Virbac, Vetflurane 1 000MG/G, cat. No. BE-V166896) **! CAUTION** volatile anesthetic agent which is administered to the mask with anesthetic spray.

- Aquasonic clear ultrasound gel (Parker Labs, Aquasonic® 100 Ultrasound Transmission Gel, ref 01-08).

Equipment: (Suppl Figure 3A-C)

a) Small material
b) Anesthetic station
c) Ultrasound machine

Procedure

1- SETUP OF THE APPARATUS • TIMING 1h at the first use

a. Training on euthanized animals

I. The pancreas is an organ whose position and limits are difficult to observe. It is just below the stomach, stretched between the two kidneys, slightly above the left curve of the spleen. To validate the location, dissection is recommended. Train your eyes to detect pancreas on wild-type animals. Perform a cervical dislocation and a dissection. Add gel and observe the pancreas without skin interference to better locate the pancreas and its specific imaging.

II. To train on animals without dissection,_1 to 2 mL of saline solution can be injected i.p., which increases the rate of optimal imaging; this procedure requires a specific ethical authorization.

b. New settings

III. The search for adapted parameters was performed and described in supplementary Table 2. To vary the parameters, use the touchscreen (including "Other Settings") and the control panel.

IV. Record the settings as a "New Preset". To configure the Aixplorer with the new settings, press the "Probe" button on the Control Panel, select the probe used, go to "New Preset", enter the new one with the Touch Screen, and confirm.

2- ANALYSIS MOUSE OF INTEREST

a. Mouse preparation • TIMING 5 min

I. Open the air and oxygen inlets that feed the MiniTag station.

II. Turn on the Minitag and the heating plate at 36 ° C, with the isoflurane flux between 2 and 3%.

III. Anesthetize the mouse of interest in the induction box, before placing it in the mask.

IV. Shave the abdomen from the middle of the sternum to the bladder down well on the sides of the mouse. Wipe the cream with a water-soaked dressing before starting the observation. ▲ CRITICAL STEP To properly observe the spleen and better locate the pancreas, which are both internal organs, shave properly the left side of the animal.

V. Apply a sufficient amount of ultrasound gel on the abdomen and on the probe. ▲ CRITICAL STEP Avoid air bubbles that would deteriorate the quality of the image. (Suppl Figure 3D,E)

b. Setting • TIMING 1 min

I. Briefly press the **ON / OFF** button located on the Control Panel. (Suppl Figure 3F, blue circle) Press the "Probe" button on the Control Panel. (Suppl Figure 3F, yellow circle). Select the SL22-7lab probe and the default setting or the new setting as described in ▸ 1-b-IV.

II. Create a new "Patient" file to record all images of the same mouse in a single folder. Press the "Patient" key on the left of the Control Panel._(Suppl Figure 3F, red circle). Enter the name of the desired "Patient".

III. Increase the gain percentage of B-mode to 34% (brighter image, Suppl Figure, 3F, B button).

IV. Zoom to 160% and focus to set the focal area deeper (-1cm). (Suppl Figure 3F, grey circle).

d. Pancreas imaging • TIMING 5-10 min

I. B-Mode - Press the "B" button and search for the pancreas and kidney. Save an image in Bmode. **? TROUBLESHOOTING**.

II. Doppler color mode - start an analysis in COL mode to avoid the localizations which contain the blood vessels. To enlarge the observation screen, use the "Select" key and then roll the TrackBall to vary the length. Decrease the Gain in COL mode, to focus on the main blood vessels. (Suppl Figure 3F, C button).

III. SWE-Mode - Press the SWE mode. Place the visualizing panel (Area) on the part of the pancreas to be analyzed and on the left kidney of the mouse. Wait for the elastography measurements to cover the entire measurement screen and then save the image. (Suppl Figure 3F, SW button). ▲ **CRITICAL STEP** Avoid pressure on the probe. **? TROUBLESHOOTING**.

IV. Then reduce the Area Size, to "Medium". Save another image.

e. Elastogram Analysis • TIMING 5 min

V. Once the series of the images are carried out, check the mouse until awake.

VI. Select the desired image on the Monitor.

V. Several measurements are possible.

1. Q-Box plot, allows freehand drawing of the measuring surface.
2. Q-Box, allows a unique measure of elastography in a predefined circle area.
3. Q-Box ratio, measures the ratio of two or more quantitative boxes.

VI. Press the "Save Image" is necessary.

VII. In B mode, measure tumoral volume.

VIII. Press the "End Exam."

IX. Select the desired patients, and export the raw image ("Export to DICOM"), images ("Export to JPEG / AVI") or measures ("Clinical data").

X. switch off the Aixplorer.

Troubleshooting

B-mode analysis

I. It sometimes happens that the pancreas is difficult to observe. The first and most recurrent reason is a swollen and filled stomach that forms a black shadow on the pancreas or even on the kidney controls. It is then necessary to find the best moment of the day to go to observe the mice in order to avoid this problem. (Suppl Figure 2D).

SWE analysis

I. The first difficulty lies in the fact that if the target organ / tumor is not measured, this is assessed by an absence of color coded SWE image superimposed to B mode image; move the probe to reach a satisfying image (Suppl Figure 2A,B).
II. If a SWE color gradient is visible outside the mouse, verify that there is a sufficient amount of gel, change the pressure exerted on the probe or its position / inclination (Suppl Figure 2C).

Supplementary Figure 1

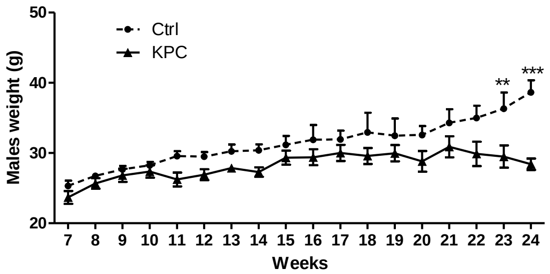

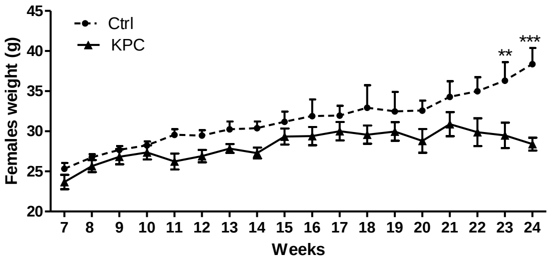

Supplementary Figure 2 **Therville N, et al**

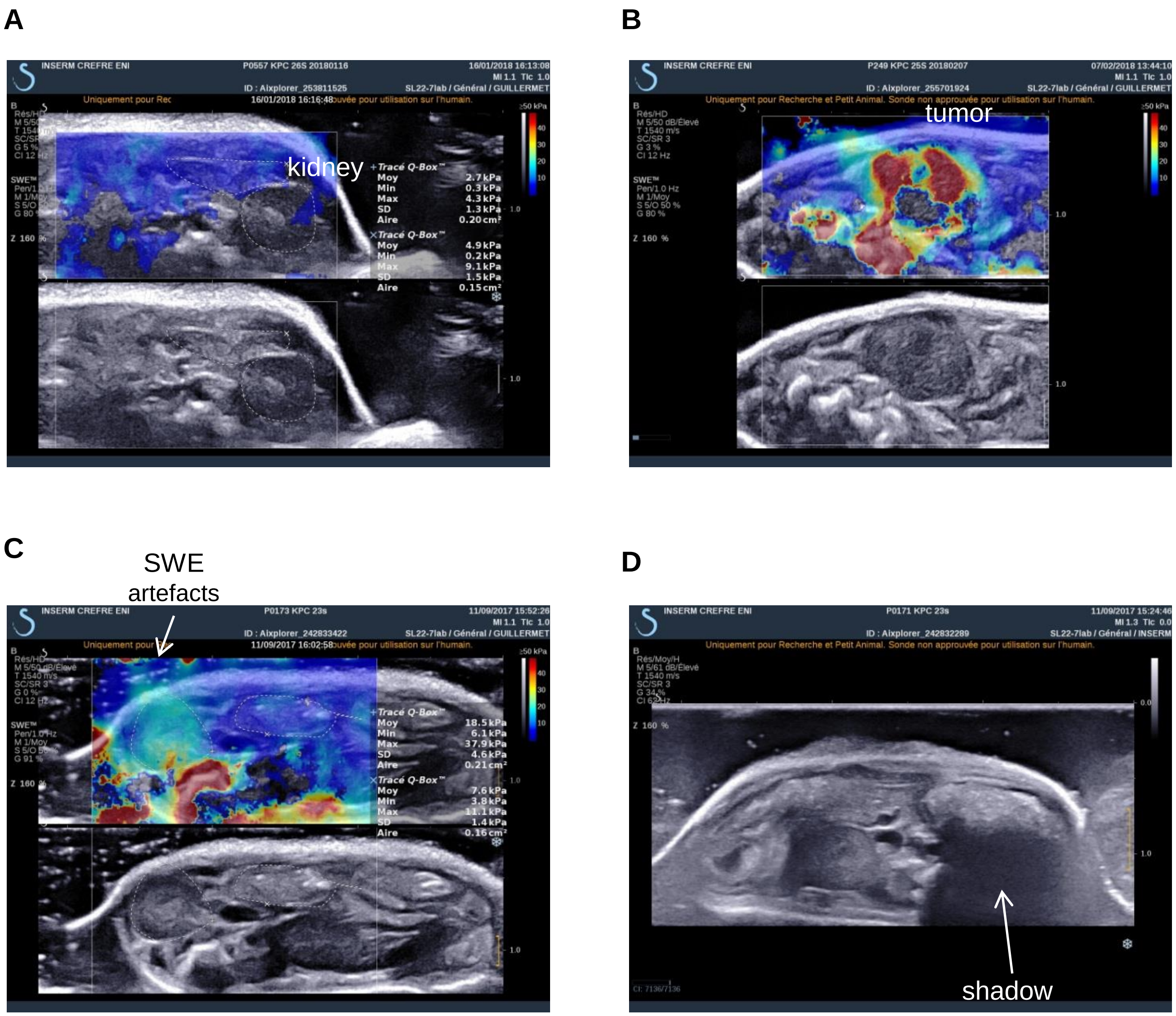

Supplementary Figure 3 Therville N, et al

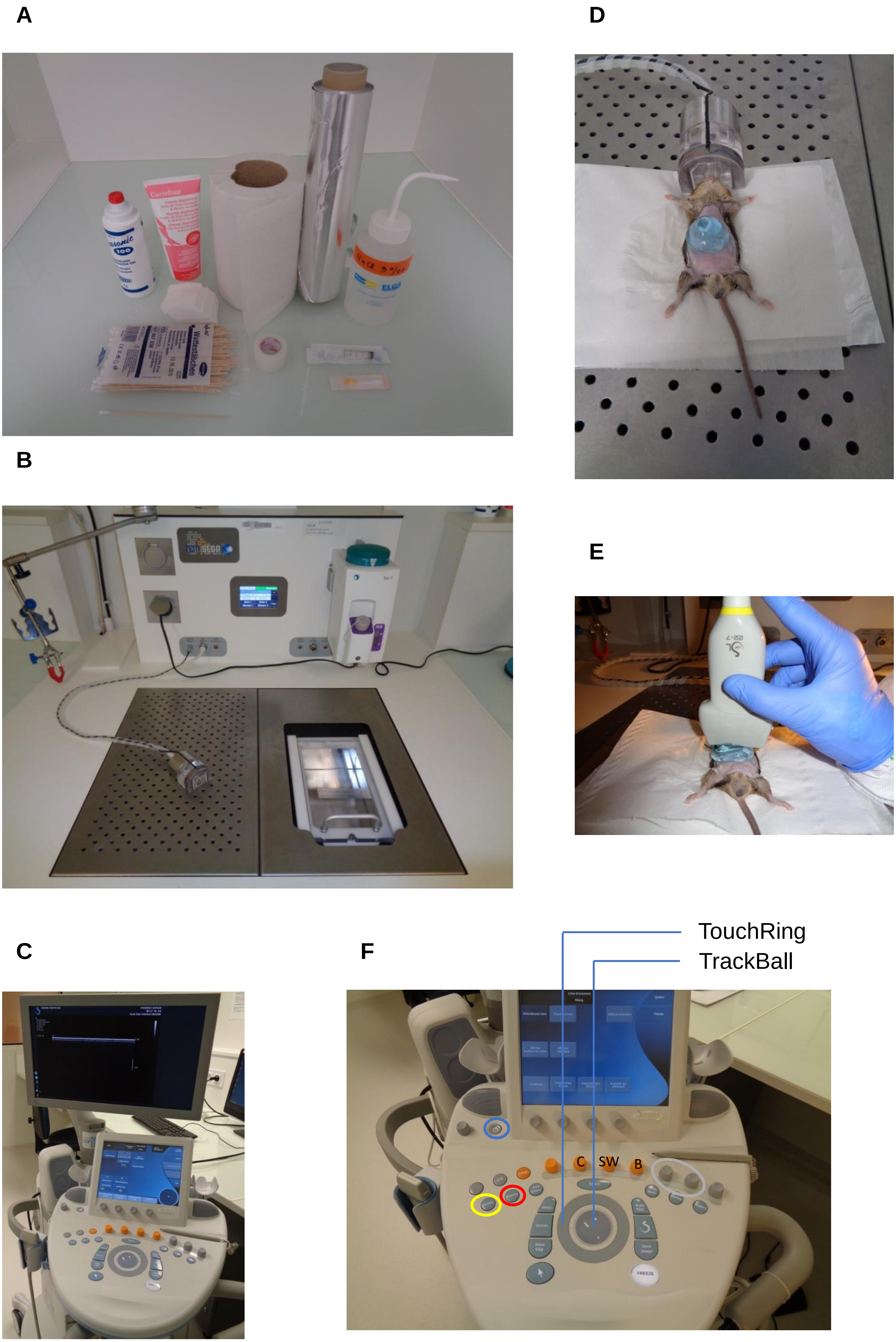

Doppler mode

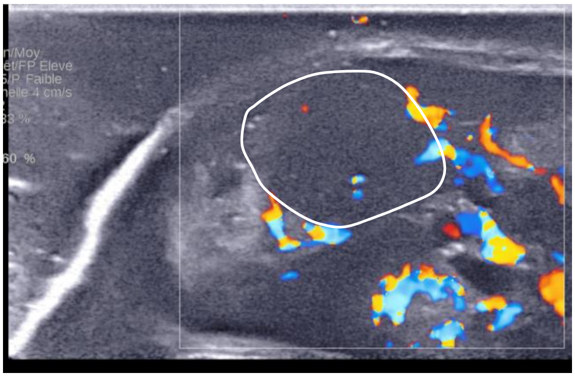

Suppl Figure 5 **Therville N. et al**

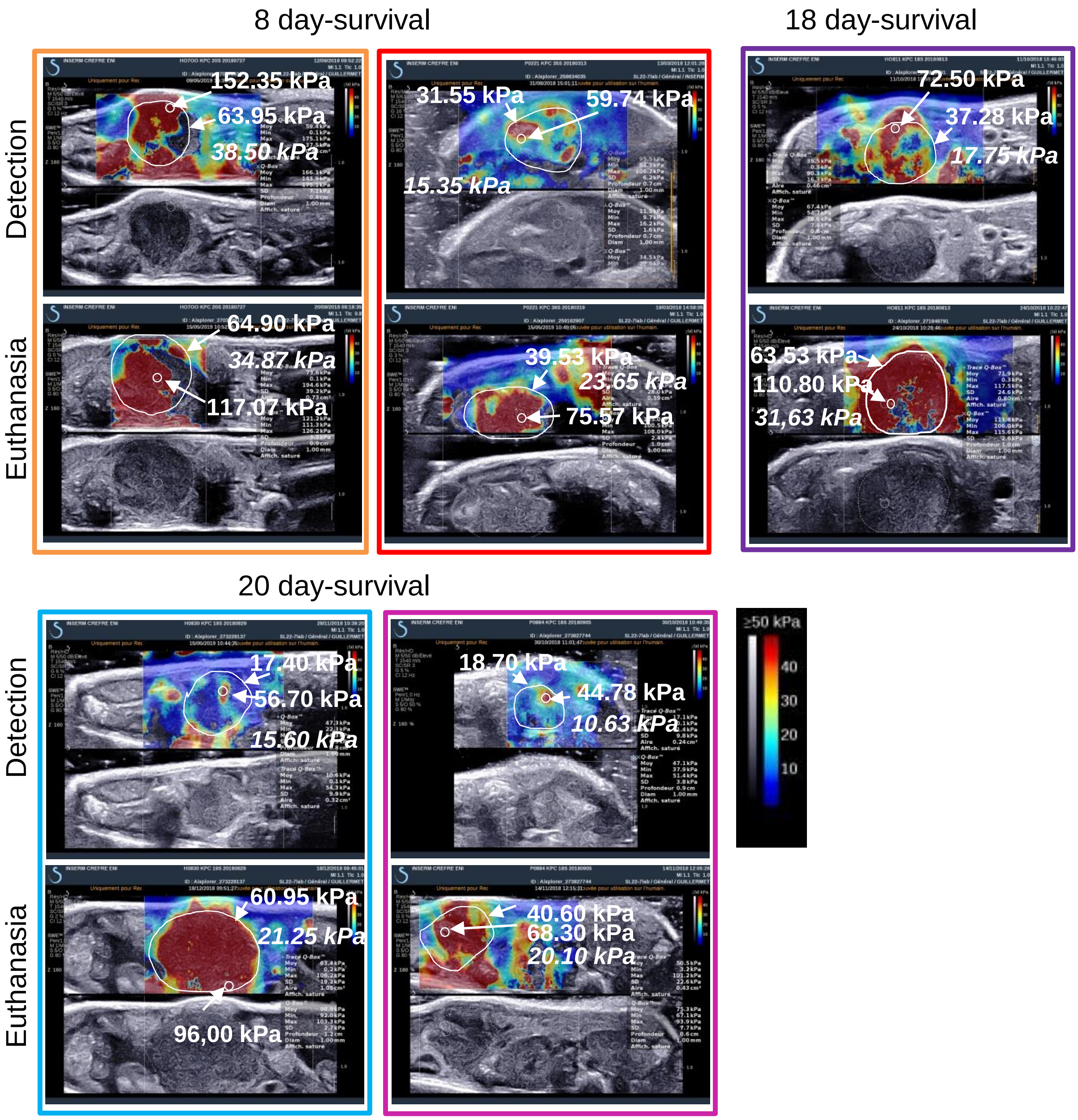

Suppl Figure 6 **Therville N, et al**

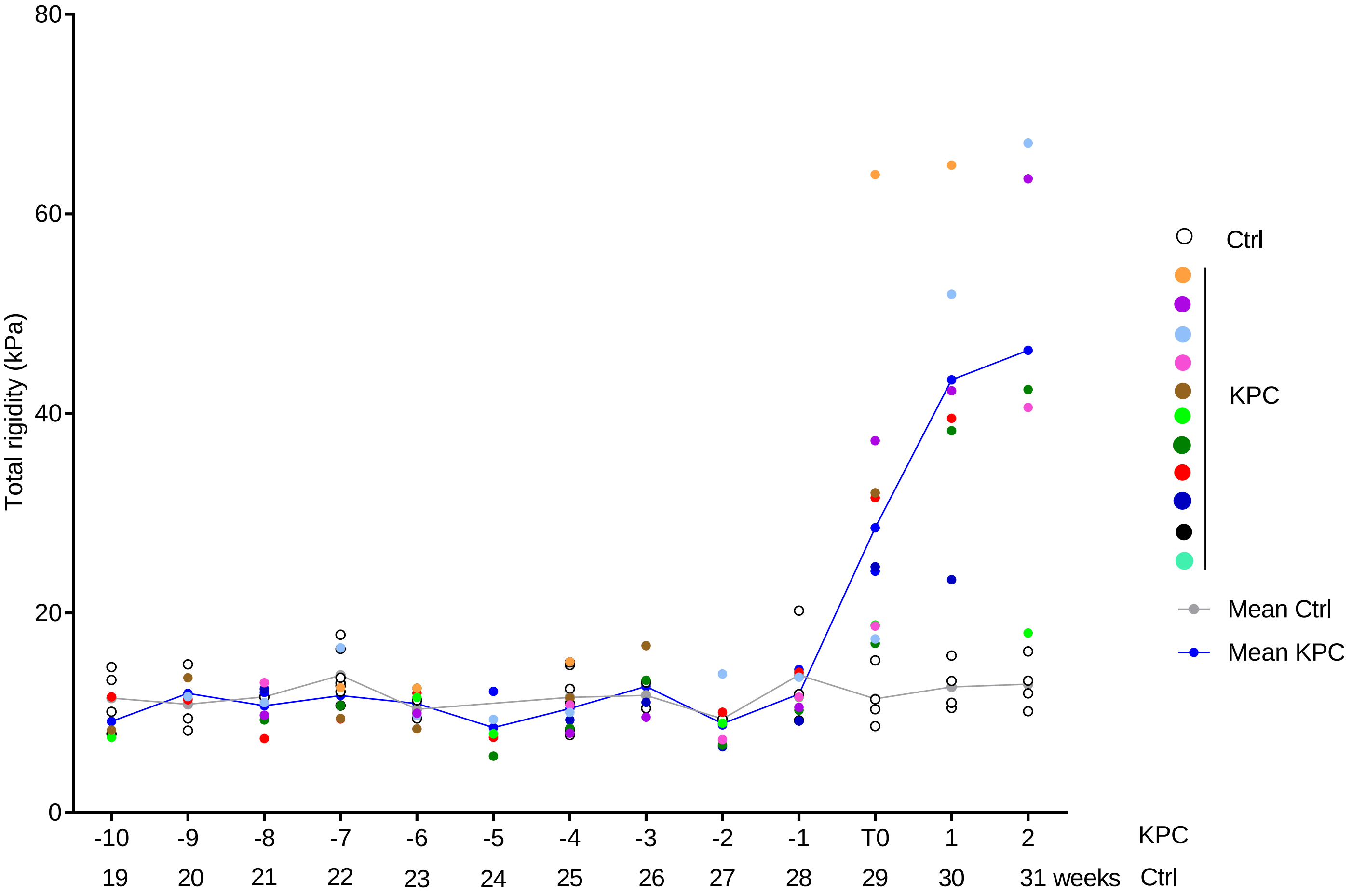

 Therville N, et al

B mode settings	Standard settings	Optimised settings	Objectives
Harmonic	yes	yes	- Reduces ultrasound artefacts and reverberation;<br>- Emphasizes contours.
SuperCompound	yes	yes	- Combines the acoustic signature of a large number of slanted lines to create an image that provides improved texture, better edge definition and reduced shadow effects.
Gain	22 %	34 %	- Adjusts the brightness of the image.
zoom	120-123%	160%	- Increases or decreases the magnification.
Resolution / General / Penetrance	Res	Res	- Adjusts the frequency center to obtain a higher resolution or penetration.
HD/Cadence Image	Intermediate	HD	- Adjusts image rate and line density to increase the spatial or temporal resolution.
Sound power	-0.5dB	-1.5dB	- Adjusts the sound power emitted by the system.
TissueTuner	1540m/s	1540m/s	- Adjusts the speed of sound according to the type of imaged tissue, which has the effect of increasing lateral resolution and contrast.
Dynamic range	61dB	50dB	- Adjusts the range of amplitudes that can be displayed.
Sector size	Great	Great – medium	- Broadens or refines the size of the area to maximize the region of interest on the image (the change in size of the sector has an impact on the image rate: the finer the sector, the better the image rate).
PRF	High	Max	- Adjusts the repetition rate of the image in B mode.
Persistence	High	High	- Temporal filter, reduces the noise on the image.
SuperRes	3	3	- Is an image processing function that reduces the flecks, improves the texture of the image and enhances the contours without changing the frame rate.
